# Letter from Louisville, Ky.

**Published:** 1882-03

**Authors:** W. Cheatham

**Affiliations:** 315 W. Walnut St., Louisville, Ky.


### ARTICLE X.

JANUARY 26, 1882.

MESSRS. EDITORS:—I see an article in your journal by Dr. Rosencrans, in reference to a case of epilepsy cured by enucleation of an eye. I think it was in 1878 I reported such a case in the *Louisville Medical News*. The woman had had fits since an injury to right eye, caused by a blow from a chip of wood. I suspected the eye as the cause, and advised an enucleation, to which she submitted, having tried all other remedies without relief. She had averaged one fit per day for several years. You can imagine her condition. I gave her ether, and while under its influence—or rather while recovering from its influence—she had ten or twelve of the most severe attacks of epilepsy I ever saw. Her head and heels almost touched. After she entirely recovered from the anæsthetic, she never had another. The "aura" appeared to her to originate in the eye. The eye was thoroughly degenerated. Synechia anterior; lens absent. Bone in choroid. Nerve with a glaucomatous excavation.

She was under my observation for two years afterward, and reported entirely well. The case was of great interest at the time to me. Have heard of several similar ones since. She took no medicine after the eye was enucleated.

Respectfully,

W. CHEATHAM, M.D.

315 W. Walnut St., Louisville, Ky.

---

OLD JOURNALS WANTED.—Dr. A. B. Judson, a gentleman of superior attainments in joint diseases, residing at No. 129 East 17th street, New York City, wishes very much to obtain the following back numbers of the CHICAGO MEDICAL EXAMINER, viz: September and December, 1860, and June, July and October, 1861. He will pay six dollars for the two volumes referred to, or one dollar each for the particular numbers above mentioned. Dr. Judson is a most worthy member of our profession, and any one able to supply him will confer a favor by writing him at the above address.

E. ANDREWS, M.D.

